# Summer Mourning for Females

**Published:** 1857-10

**Authors:** Frederick J. Brown


### ON SUMMER MOURNING FOR FEMALES.

By FREDERICK J. BROWN, M.D.

The higher temperature of dark-coloured than of light-coloured clothes, when exposed to the rays of the sun, is a fact well known to the public. It is in consequence of the appreciation of this difference in the effects of colour, that we see so many persons dressed in light-coloured garments during the summer months. It is desirable that change of colour in articles of dress, according to season, should become the custom with men as well as with women, instead of being exceptional as it is at present. The black hat, with the black cloth clothes, cause men to experience distress that is wholly unnecessary. The unwritten laws of society are binding upon civilized men, but they must not be carried out to the extent of inflicting useless suffering. Men living in communities, however, do rightly submit to rules of conduct, and also to rules relating to dress. It is proper, therefore, to change the public customs rather than to liberate men from the control of public opinion.

It is common for the observation to be made by those that have returned from warm climates, that the heat in England, in the summer, is less patiently borne than it is in hot countries. This circumstance is due to the insufficient difference that is made betwixt winter and summer clothing, particularly as regards colour. The natives of hot countries commonly wear white or light-coloured articles of dress in the hot season, and they practise every method that is available to nullify the effects of solar heat upon their bodies; and Europeans on the continent follow their example to some extent. In England, on the contrary, great pains are taken to guard against the effects of cold; and the hot weather is considered by the public as something good because it is opposed to "cold."

The proper course to follow is to moderate the effects of cold in the winter, and of heat in the summer. It is well known that both cold and heat in excess are injurious to health.

The tables of mortality show an increase in the number of deaths from pulmonary affections when the thermometer falls in winter, and an augmentation in the mortality of diarrhœa when the temperature rises high in summer; and the increase corresponds accurately, allowing for disturbing causes, with the fall or rise of the thermometer respectively.

Women readily exchange their winter garments for those suitable to summer; but, under circumstances of mourning, they are cruelly compelled by custom to move about under a load of black crape. It is to liberate them from this misery that the present article is written.

Many widows suffer from nervous headache in consequence of night-watching, anxiety, and grief; and this form of headache is converted into congestion of the blood-vessels of the head by exposure to the sun in black bonnets and dresses. There are numerous instances of widows remaining within doors for months together, to the great injury of their health, rather than endure the misery of sun broiling.

The remedy is very simple.

Let summer mourning become customary. Let light-coloured clothing be worn, trimmed with thin black edging.

There is such an article as white crape; but it indicates slight mourning. Either white crape should be worn as summer mourning, or small-sized black edging to light-coloured dresses; and bonnets should be introduced into general use for the purpose.

The milliners can best manage the details: it is for physicians to point out the propriety of the change in custom.

Chatham, September 1857.

